# Unusually large exciton binding energy in multilayered 2H-MoTe_2_

**DOI:** 10.1038/s41598-022-08692-1

**Published:** 2022-03-16

**Authors:** Eilho Jung, Jin Cheol Park, Yu-Seong Seo, Ji-Hee Kim, Jungseek Hwang, Young Hee Lee

**Affiliations:** 1 Center for Integrated Nanostructure Physics (CINAP), Institute for Basic Science (IBS), Sungkyunkwan University (SKKU), Suwon, 16419 Republic of Korea; 2 Department of Physics, Sungkyunkwan University (SKKU), Suwon, 16419 Republic of Korea; 3 Department of Energy Science, Sungkyunkwan University (SKKU), Suwon, 16419 Republic of Korea

**Keywords:** Materials science, Physics

## Abstract

Although large exciton binding energies of typically 0.6–1.0 eV are observed for monolayer transition metal dichalcogenides (TMDs) owing to strong Coulomb interaction, multilayered TMDs yield relatively low exciton binding energies owing to increased dielectric screening. Recently, the ideal carrier-multiplication threshold energy of twice the bandgap has been realized in multilayered semiconducting 2H-MoTe_2_ with a conversion efficiency of 99%, which suggests strong Coulomb interaction. However, the origin of strong Coulomb interaction in multilayered 2H-MoTe_2_, including the exciton binding energy, has not been elucidated to date. In this study, unusually large exciton binding energy is observed through optical spectroscopy conducted on CVD-grown 2H-MoTe_2_. To extract exciton binding energy, the optical conductivity is fitted using the Lorentz model to describe the exciton peaks and the Tauc–Lorentz model to describe the indirect and direct bandgaps. The exciton binding energy of 4 nm thick multilayered 2H-MoTe_2_ is approximately 300 meV, which is unusually large by one order of magnitude when compared with other multilayered TMD semiconductors such as 2H-MoS_2_ or 2H-MoSe_2_. This finding is interpreted in terms of small exciton radius based on the 2D Rydberg model. The exciton radius of multilayered 2H-MoTe_2_ resembles that of monolayer 2H-MoTe_2_, whereas those of multilayered 2H-MoS_2_ and 2H-MoSe_2_ are large when compared with monolayer 2H-MoS_2_ and 2H-MoSe_2_. From the large exciton binding energy in multilayered 2H-MoTe_2_, it is expected to realize the future applications such as room-temperature and high-temperature polariton lasing.

## Introduction

One of the key features of strong Coulomb interaction in two-dimensional (2D) van der Waals (vdW) layered materials is large exciton binding energy and spin–orbit coupling (SOC) in the monolayer TMDs^1,2^. Information on optical bandgap and exciton binding energy of a material is a primary concern for various applications in electronic, optical/optoelectronic, and photocatalytic devices^3–5^. Large exciton binding energy, particularly in a monolayer, can be attributed to neutral excitons as well as multi-excitons, such as trions and biexcitons, at room temperature^6–9^. Moreover, the large exciton binding energy can be used for room-temperature polariton lasing and heterostructure physics such as interlayer exciton^10–12^. The SOC in 2D transition metal dichalcogenide (TMD) materials originating from the *d*-orbitals of transition metals breaks inversion symmetry, splitting the valence band around K-point^13,14^. TMD materials with broken inversion symmetry are promising for applications applied to spin-valleytronics, in which their spin degree of freedom and valley polarization can be manipulated^15^. Despite the possibilities of a various applications, it is hard to apply in reality. Because material values such as bandgap, exciton binding energy, and SOC energy of TMD materials are stochastically scattered from material to material, let alone measurement tools.

In monolayer TMDs, the atoms are fully exposed to vacuum and involve strong Coulomb interaction and reduced dielectric screening effect, consequently exhibiting, in general, large exciton binding energies of typically 0.45–1.04 eV^16–20^, which are distinct by one order of magnitude from the lower exciton binding energies (2.7–40 meV) of 3D semiconducting materials such as Si, Ge, and GaAs^21–23^. The exciton binding energy strongly relies on materials. For example, monolayer 2H-MoS_2_ and 2H-WS_2_ exhibit relatively large exciton binding energies of 640–830 meV^19,24^, whereas some values are as small as ~ 160 meV in 2H-WS_2_^25^. Such a large variance can be attributed to substrate effect, charge transfer, strain, and residual adsorbents under ambient conditions. Nevertheless, strong material dependence of exciton binding energy has been reported in the literatures.

The exciton binding energy in multilayered TMDs has not been sufficiently explored. In general, the exciton binding energies in multilayered TMDs vary with the thickness of the material as well as the atomic masses of the constituent atoms. The exciton binding energy decreases with increasing thickness of the material due to increased screening, whereas it decreases when the chalcogen atom is changed from the light S to the heavy Te^16^. Nevertheless, the exciton binding energies of bulk TMDs are still higher than those of 3D semiconducting materials. For example, the energies in the TMDs in 2H-MoS_2_ bulk^26,27^ are as high as 50–84 meV and approximately 150 meV in 2H-MoTe_2_ bulk^28^, and as low as 67 meV in 2H-MoSe_2_ bulk and 50 meV in 2H-WSe_2_ bulk^26^. It should be noted that the lower bound of binding energies is still higher than those (3–10 meV) of bulk materials^21–23^. This implies that the origin of large exciton binding energy in TMD materials does not simply rely on the thickness. The binding energy is further complicated by involving the SOC, dielectric constant and Coulomb interaction of the material.

The exciton binding energy and bandgap can be directly measured by combining optical absorption and/or photoluminescence (PL) with photocurrent spectroscopy or scanning tunneling spectroscopy (STS)^29–31^. Theoretical calculation of the quasi-particle bandgap may allow to estimate the exciton binding energy. The difficulty arises owing to the uncertainty caused by the two combined experimental systems or inaccurate estimation of bandgap from theory. For example, the measurements of exciton binding energy and bandgap in TMDs vary according to the different measurement systems used, such as PL combined with photocurrent, PL/STS, and PL/density functional theory (DFT)^19,29–33^. The reflectance/transmittance contrast spectrum is introduced to measure the exciton energy without explicitly measuring the bandgap^28,34–37^. Therefore, a robust measurement system is required to accurately measure the exciton energy and bandgap.

In this study, we report the thickness- and temperature-dependent optical properties of few-layered 2H-MoTe_2_ thin films grown by chemical vapor deposition (CVD). Fourier-transform infrared (FTIR) spectroscopy is used to measure the transmittance spectra of semiconducting 2H-MoTe_2_ thin films with various thicknesses 2 (3 layers), 4 (6 layers), and 10 (14 ~ 15 layers) nm, and their optical conductivities are obtained using a thin film analysis method, which is known as a transfer matrix method^38,39^. The optical conductivities are analyzed using the symmetric Lorentz and asymmetric Tauc–Lorentz models^40–42^ to extract information on direct/indirect bandgaps and exciton binding energy. We obtain significantly large exciton binding energy of around 300 meV at the K- and Γ-points in the Brillouin zone in multilayered 2H-MoTe_2_ compared to those of other TMDs with similar thicknesses. Our results provide detailed optical and electronic information on few-layered 2H-MoTe_2_ thin films, which can be applied to optoelectronic devices such as solar cells, detector, and polariton lasing.

## Result and discussion

**Figure 1:**
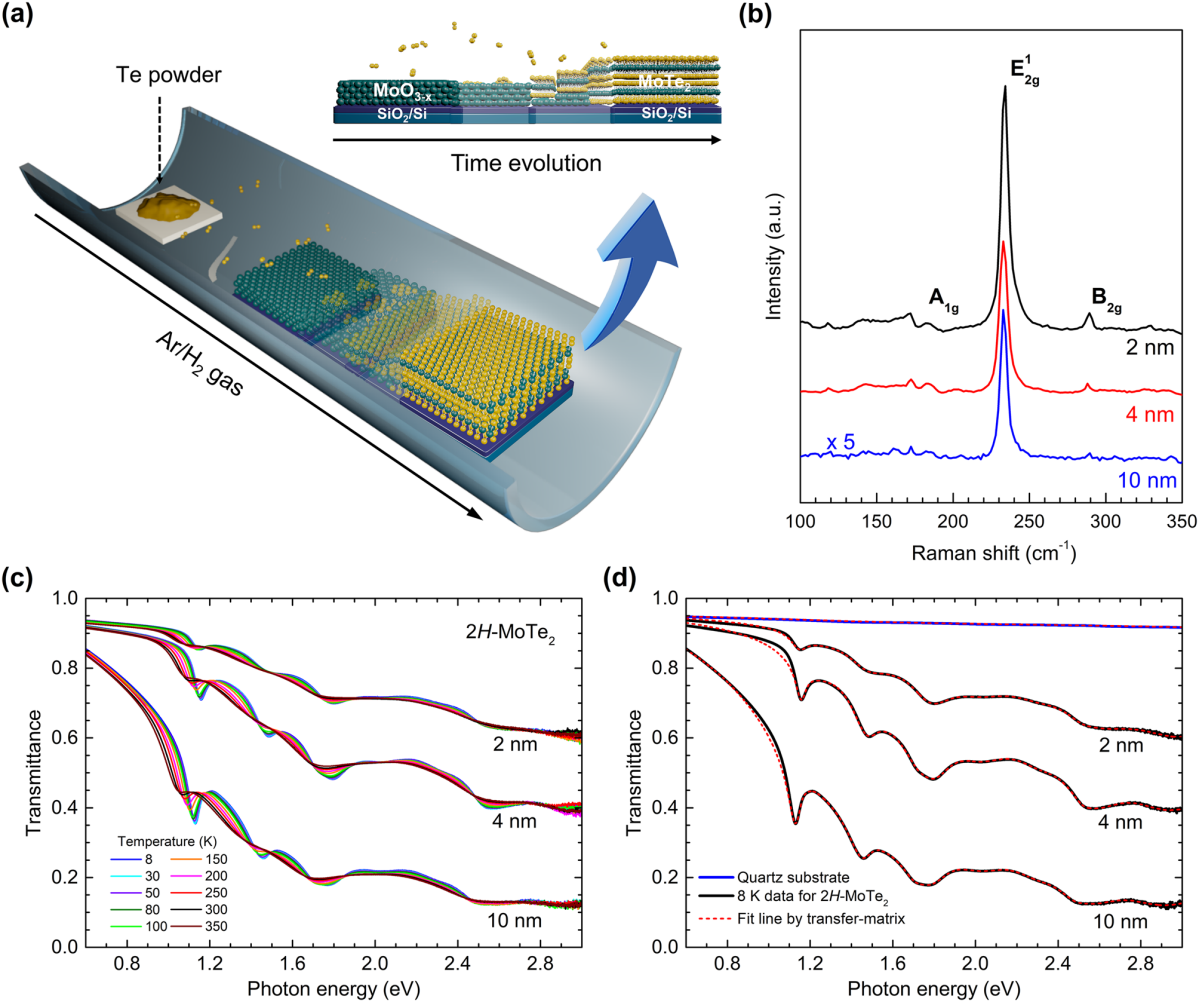
Synthesis of 2H-MoTe_2_ film and measurement transmittance spectra. (**a**) Schematic of chemical vapor deposition system used to synthesize 2H-MoTe_2_ film on SiO_2_/Si substrate and illustration of growth process. The oxidized Mo thin film transformed gradually to the 2H-MoTe_2_ film as the processing time goes. (**b**) Thickness-dependent Raman spectra with three different thicknesses (2, 4, and 10 nm). (**c**) Measured transmittance spectra for 2H-MoTe_2_ films at various temperatures between 8 and 350 K. (**d**) Measured transmittance spectra of the three samples and their simulated spectra by using the transfer matrix method at 8 K. The transmittance and fit of quartz substrate are also displayed.

The 2H-MoTe_2_ films were synthesized using the CVD method with Mo thin films and Te pellets as Mo and Te sources, respectively. Figure 1a shows the schematic of a CVD system along with the growth process (the details are provided in Methods). Three different thicknesses of the samples (2, 4, and 10 nm; Figure S1, Supplementary Information) were characterized using Raman spectroscopy with a laser of wavelength 532 nm. The characteristic Raman active modes of A_1g_ (~ 173 cm^−1^), E^1^_2g_ (~ 235 cm^−1^), and B^1^_2g_ (~ 290 cm^−1^) were clearly observed; these were independent of thickness beyond 2 nm. The B^1^_2g_ mode appeared for thin samples, which is absent in monolayer 2H-MoTe_2_ (Fig. 1b)^43^. The intensity of the B^1^_2g_ mode reduces with increasing thickness; it can be used as a measure for the thickness of the 2H-MoTe_2_ thin film^43^. In addition, 2H-MoTe_2_ films studied in this work have been characterized by using Raman mapping, X-ray diffraction (XRD), transmission electron microscopy (TEM) in previous report^44^. The samples were polycrystalline, and the crystallinity was 10 μm or more.

The 2H-MoTe_2_ films are prepared on a 6 × 6 mm^2^ quartz substrate for optical investigation, and the diameter of the optical probe beam is approximately 4 mm. The measured transmittance spectra of the three samples are shown in Fig. 1c. The transmittance increases as the 2H-MoTe_2_ film becomes thinner. The optical conductivities of the 2H-MoTe_2_ films were extracted from the measured transmittance spectra using a transfer matrix method, which is a rigorous method including multiple reflections in both film and substrate^38,39^. A more detailed description how we obtained the optical conductivity from the measured transmittance can be found in Supplementary Information. Representative data at 8 K and fits using the transfer matrix are shown in Fig. 1d.

**Figure 2:**
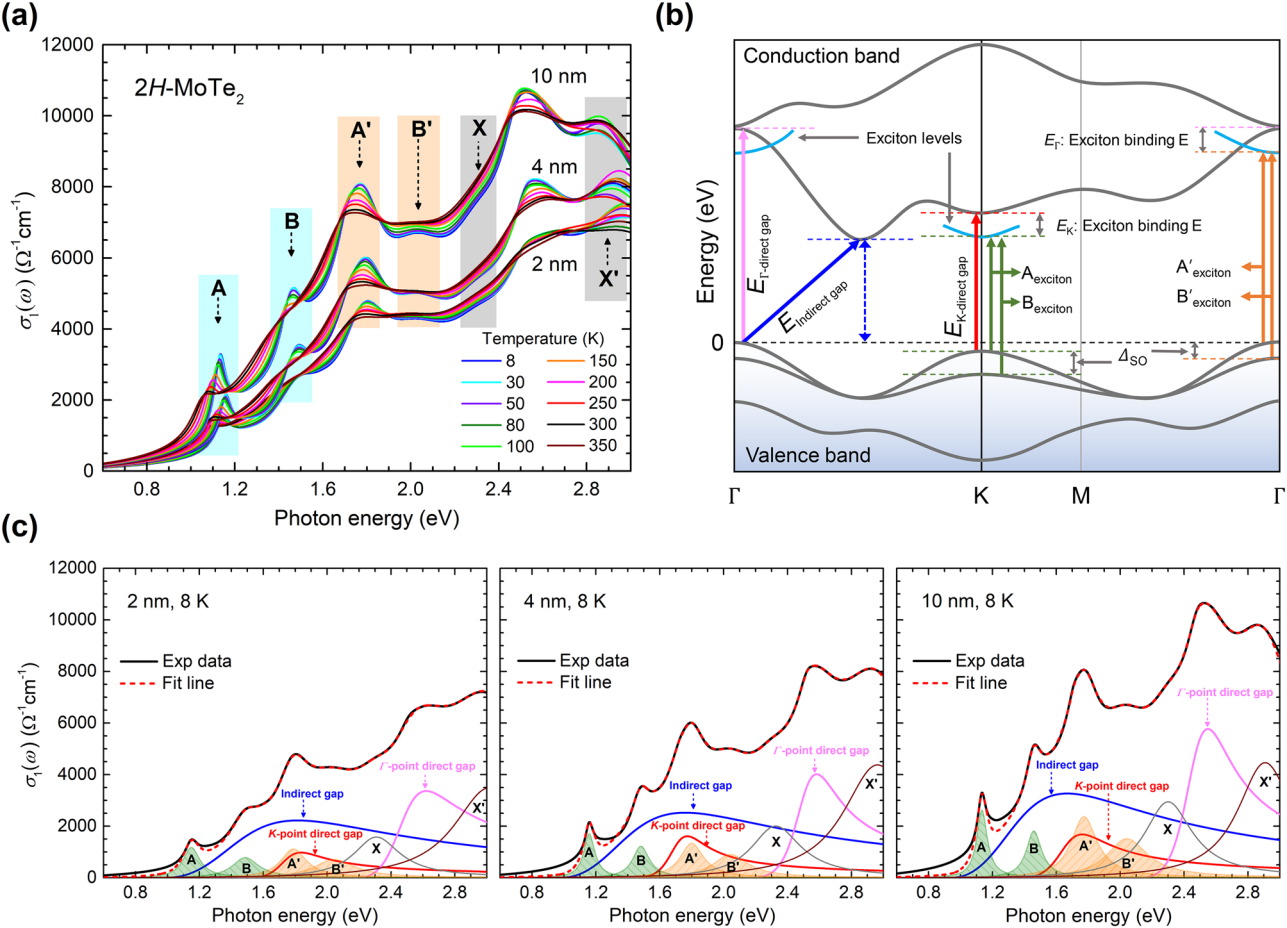
Analysis of real part of optical conductivity and optical designation in band structure of TMDs. (**a**) Real part of optical conductivities for 2, 4, and 10 nm thick 2H-MoTe_2_ at various temperatures between 8 and 350 K. (**b**) Schematic of band structure of multilayered TMDs with optical designations. (**c**) Real part of optical conductivities fitted using Lorentz and TL models at 8 K.

Figure 2a shows the optical conductivities of 2H-MoTe_2_ thin films at various temperatures and thicknesses 2, 4, and 10 nm. The well-known excitonic absorption peaks have shifted to the low-energy side and broadened with elevating temperature. Each exciton peak is clearly distinguished at low temperature. The optical conductivity in Fig. 2a is directly related to the electronic band structure of the material. The optical conductivity reflects all possible transitions from filled states below the Fermi level to empty states above it and is a function of only energy or frequency, i.e., the momentum averaged quantity over the Brillouin zone. Figure 2b shows the schematic of the band structure with conduction and valence bands in typical multilayered TMDs^16,45–47^. A direct bandgap appears at the K- (smaller *E*_g_) and Г- (larger *E*_g_) point, and the smallest indirect bandgap appears at the intermediate 0.52 Г-K point^16^. We note that the indirect transition can be possible by other processes which hold the momentum conservation law such as phonon-assisted process^48^. One prominent feature in TMD materials is the existence of the large SOC energy, giving rise to the splitting of energy bands, which is distinct from the case of small SOC in 3D materials^16,26,46^. As the SOC energy at the conduction band edge is relatively low compared to that at the valence band edge, the SOC energy at the conduction band edge is typically ignored in TMD materials. Exciton peaks are assigned as A and B near the K-point and Aʹ and Bʹ near the Г-point. It should be noted that the exciton absorption bands are symmetric, in contrast with asymmetric bandgap absorptions.

In this study, we fully analyze symmetric and asymmetric absorption band structures in the optical conductivity using two models: Lorentz and Tauc–Lorentz (TL) models. The Lorentz model can be used to describe symmetric exciton absorptions (electron–hole pairs), whereas the TL model can be used to describe asymmetric direct and indirect bandgap absorptions^40–42^. The real part of optical conductivity can be described using the Lorentz and TL models as ${\sigma }_{1}\left(\omega \right)=\frac{1}{4\pi }{\sum }_{i}\frac{{\gamma }_{\mathrm{i}}{\omega }^{2}{A}_{\mathrm{i}}}{{\left({\omega }_{\mathrm{i}}^{2}-{\omega }^{2}\right)}^{2}+{\gamma }_{\mathrm{i}}^{2}{\omega }^{2}}+\frac{1}{4\pi }{\sum }_{j}\frac{{B}_{\mathrm{j}}{\omega }_{\mathrm{j}}{\gamma }_{\mathrm{j}}{\left({\omega -E}_{\mathrm{g},\mathrm{j}}\right)}^{2}}{{\left({\omega }^{2}-{\omega }_{\mathrm{j}}^{2}\right)}^{2}+{\gamma }_{\mathrm{j}}^{2}{\omega }^{2}}\Theta (\omega -{E}_{\mathrm{g},\mathrm{j}})$, where ${A}_{\mathrm{i}}$, ${\omega }_{\mathrm{i}}$, and ${\gamma }_{\mathrm{i}}$ are the oscillator strength, resonance frequency, and damping parameter of the $i$ th Lorentz component, respectively and ${\omega }_{\mathrm{j}}$, ${E}_{\mathrm{g},\mathrm{j}}$, ${B}_{\mathrm{j}}$ and ${\gamma }_{\mathrm{j}}$ are the resonance frequency, bandgap (or absorption edge), oscillator strength, and damping parameter of the $j$th Tauc–Lorentz component, respectively, and $\Theta (x)$ is the Heaviside step function. Here, the Drude component is not included since the systems are undoped semiconductors. For our multilayered TMD materials, four symmetric Lorentz components (A, B, A’, and B’) and three asymmetric TL components (indirect and direct bandgaps at the K- and Г-points) are included to fit the optical conductivity^36,49,50^. It is worth noting that the Г-point direct bandgap has been assigned as C mode in previous literature^36,49^.

**Figure 3:**
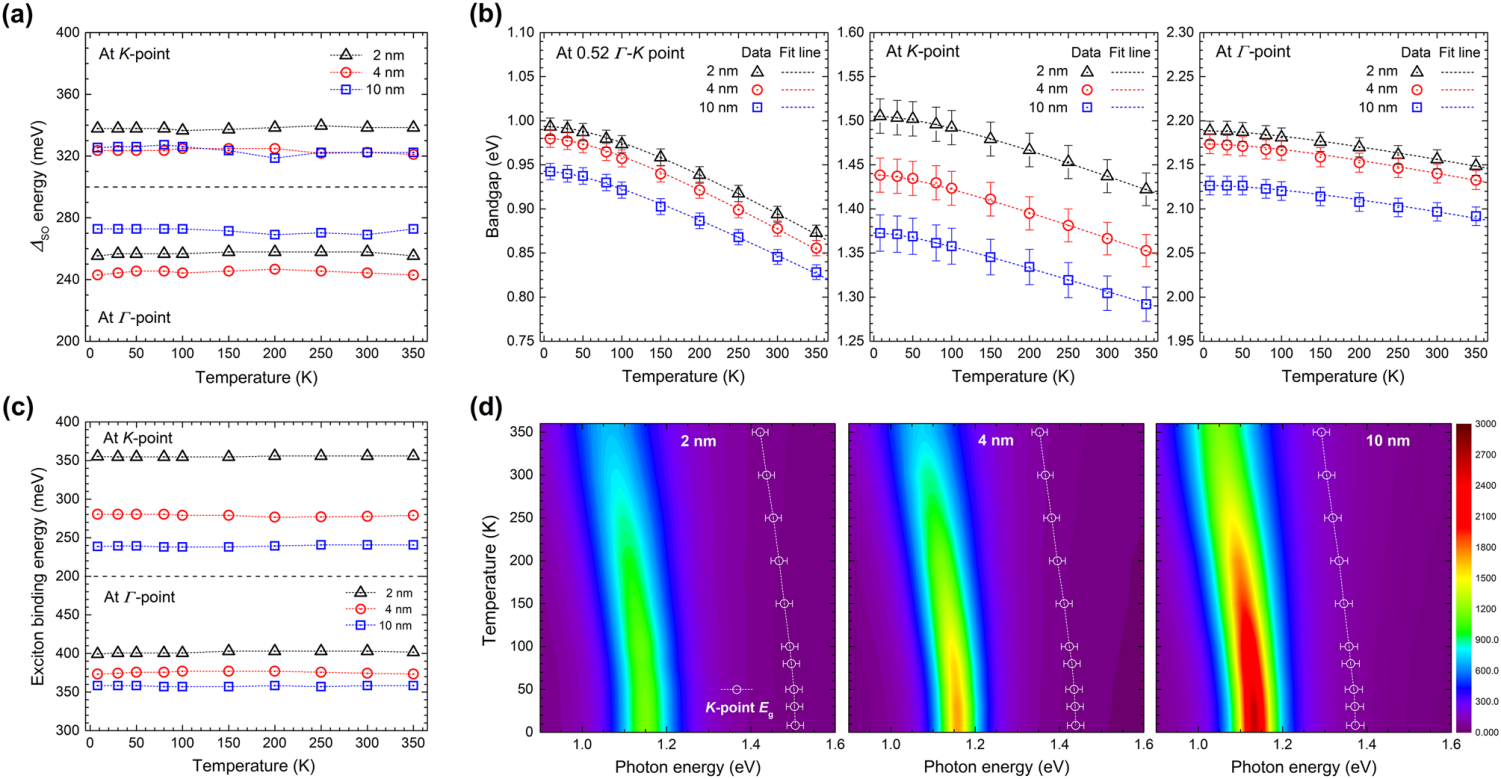
Fitting parameters extracted using Lorentz and TL models. (**a**) *Δ*_SO_ at K- and Γ-point for 2, 4, and 10 nm thick 2H-MoTe_2_ at various temperatures between 8 and 350 K. (**b**) Temperature- and thickness-dependent indirect and direct bandgaps of 2H-MoTe_2_ obtained using Lorentz and TL models. The fitting lines are calculated using temperature-dependent bandgap equation. (**c**) Exciton binding energy at K- and Γ-point for 2, 4, and 10 nm thick 2H-MoTe_2_ at various temperatures between 8 and 350 K. (**d**) Color maps (${\sigma }_{1}$) of the A exciton and the direct bandgap edge at K-point as functions of photon energy and temperature.

The observed conductivity with 2, 4, and 10 nm thick 2H-MoTe_2_ at 8 K is deconvoluted using the aforementioned Lorentz and TL components (Fig. 2c) (see Supplementary Information Figures S2, S3, and S4 for additional fittings at various temperatures between 8 and 350 K). The peak positions (${\omega }_{i})$ of four Lorentz components are extracted directly from the conductivity spectra with approximate spectral widths (${\gamma }_{j}$). The optical conductivity is deconvoluted using additional asymmetric components (two direct bandgaps at the K- and Г-points and an indirect bandgap at 0.52 Г-K point); which is well fitted below 2 eV (see Supplementary Information Figure S5). These absorption modes including the prominent exciton modes are based on the calculated electronic band structure shown in Fig. 2b. Beyond 2 eV, the fitting is further improved by adding two unknown Lorentz components (X and X’). We note that X and X’ are not new; they were observed and denoted as ϕ and D in previous literature^49^. As far as we know, these two modes have not been clearly identified yet. It is also worth noting that we needed an additional weak Lorentz mode between A and B excitons. We think that this additional mode can be assigned as either the 2 s-like excited state of the exciton A or the ground state interlayer exciton, which were observed in a previous report^28^. The additional mode shows temperature-dependence; it becomes weaker as temperature increases (see Figures S2, S3 and S4 in the Supplementary Information). We show the temperature-and thickness-dependent width and position of A and B excitons of 2H-MoTe_2_ in Supplementary Information (see Supplementary Information Figure S6). The temperature-dependent positions of A and B excitons of MoTe_2_ are similar to reported results of WS_2_ and WSe_2_^24^. The additional mode also shows thickness-dependence; it becomes stronger as the thickness increases (see Supplementary Information Figure S7). We also took the second derivative of the measured transmittance spectra to check any small features related to excitation states near the excitons and found a feature just above the peak energy of the exciton A, which might be related to the 2 s state. However, we could not see any higher excited states (see Supplementary Information Figure S7). The extracted spin–orbit splitting band energy (*Δ*_SO_), bandgap, and exciton binding energy are summarized in Fig. 3.

Figure 3a shows the thickness- and temperature-dependent *Δ*_SO_ at the K- and Γ-point of 2H-MoTe_2_ with thicknesses of 2, 4, and 10 nm. The value of *Δ*_SO_ at the K-point is 335.4 meV for the 2 nm sample at 8 K; it is mostly independent of the temperature range of 8–350 K and slightly lower for thicker samples. The value of *Δ*_SO_ at the Γ-point for the 2 nm sample at 8 K is 262.9 meV, lower than that at the K-point, although its dependence on temperature and thickness are not evidently distinct. The bandgap decreases with temperature (Fig. 3b), similar to 3D bulk semiconductors. The indirect bandgap for the 2 nm 2H-MoTe_2_ at low temperature (8 K) is 0.96 eV; it reduces further to 0.87 eV at 10 nm, similar to that (0.88 eV) for bulk 2H-MoTe_2_^51^. The dashed lines are fitting lines based on the temperature-dependent bandgap equation, ${E}_{\mathrm{g}}\left(T\right)= {E}_{\mathrm{g}}\left(0\right)- \frac{\alpha {T}^{2}}{T+\beta }$ , where ${E}_{\mathrm{g}}\left(0\right)$ is the bandgap at $T$ = 0, $\alpha$ and $\beta$ are fitting parameters. It can be seen in Table 1 that the values of $\alpha$ and $\beta$ are lower than those of indirect bulk materials such as Si and Ge. Fitting parameters of $\alpha$ and $\beta$ decrease as increasing the thickness for the indirect bandgap. In contrast, the fitting parameter $\beta$ increases when the thickness increases for the direct (K-point) bandgap. Otherwise, fitting parameters do not follow the trends of the indirect and direct (K-point) bandgaps for the direct (Γ-point) bandgap. Furthermore, the extent of change is not proportional to the thickness. For the 2 nm sample, a relatively large direct bandgap of 1.5 eV appears at 8 K at the K-point, reducing to 1.39 eV for the 10 nm sample. The bandgaps are further reduced with temperature, similar to the indirect bandgaps. The variance in the direct bandgap at the Γ-point with thickness and similar fitting parameters is nearly negligible.

**Table 1:** Parameters of temperature-dependent bandgap.

	Si	Ge	GaAs	2H-MoTe_2_								
				Indirect			Direct (K-point)			Direct (Γ-point)		
				2 nm	4 nm	10 nm	2 nm	4 nm	10 nm	2 nm	4 nm	10 nm
*E*_g_ (0) [eV]	1.166^56^	0.744^56^	1.519^56^	0.993	0.980	0.943	1.505	1.438	1.373	2.188	2.174	2.126
*α* [10^–4^ eV/K]	4.73^56^	4.77^56^	5.41^56^	3.70	3.50	2.70	3.40	3.50	3.60	3.80	3.90	4.50
*β* [K]	636^56^	235^56^	204^56^	145	125	90.5	110	220	290	740	540	400

The exciton binding energy at the K-point for the 2-nm-thick 2H-MoTe_2_ at 8 K is 355 meV; it reduces to 238 meV for the 10-nm-thick 2H-MoTe_2_ at the same temperature. The variance in temperature is nearly negligible. The exciton binding energy at the K-point is markedly large compared to previous reports^28,52^. Supplementary Information Tables S1 and S2 illustrate that the exciton binding energies of most TMD materials, such as 2H-MoS_2_, 2H-MoSe_2_, and 2H-WSe_2_, are in the range of 40–84 meV, one order of magnitude lower than that of the thin 2H-MoTe_2_ film. The exciton binding energy at the Γ-point is approximately 370 meV, almost independent of the sample thickness. Color maps (${\sigma }_{1}$) of the A exciton intensities as functions of photon energy and temperature for various thicknesses are represented in Fig. 3d. Here, we clearly observe almost temperature-independent exciton binding energies for all three 2H-MoTe_2_ film samples (see Fig. 3c) since both the peak position of exciton A and the direct bandgap edge at K- point show almost the same temperature-dependence (see Fig. 3d). The error bars in the bandgap edge curves are the confidence levels of the bandgap edge.

**Figure 4:**
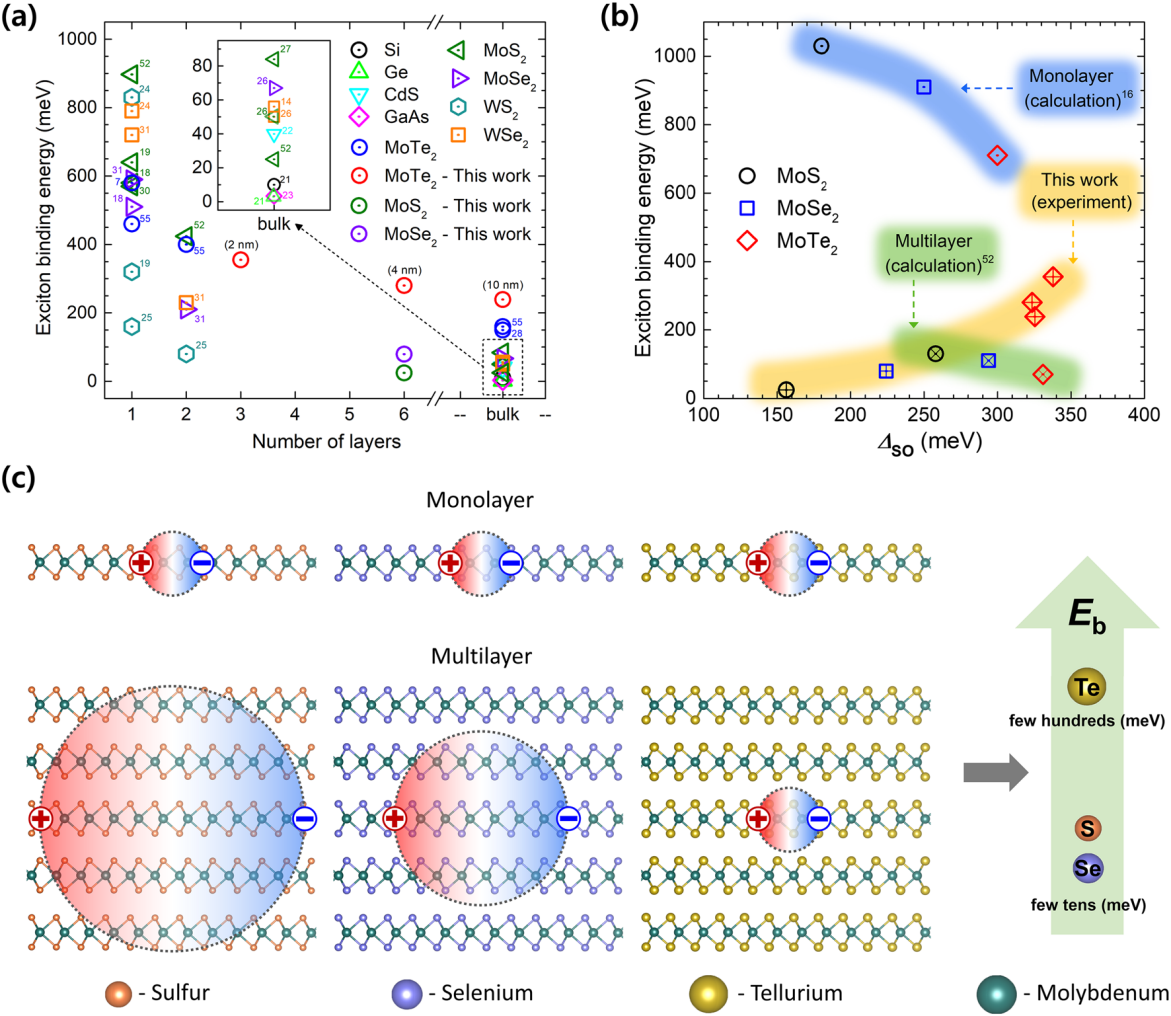
Unusually large exciton binding energy in multilayered 2H-MoTe_2_. (**a**) Comparison of exciton binding energy with diverse semiconducting materials as function of thickness. Exciton binding energies were taken from previous reports ^7,14,18,19,21–28,30,31,52,55^. (**b**) Comparing exciton binding energy of MoX_2_ as a function of *Δ*_SO_. Calculated exciton binding energies of monolayer and multilayered MoX_2_ were taken from previous reports ^16,52^. (**c**) Schematics of possible scenarios for the exciton radii in screening effect and large exciton binding energy in multilayered 2H-MoTe_2_.

Thus far, we have learned from the absorption spectra that the exciton binding energy of multilayered 2H-MoTe_2_ is approximately 238–355 meV, which is unusually large, contrary to general belief, compared to the values of 24.8–79.4 meV obtained for multilayered 2H-MoS_2_ and 2H-MoSe_2_. Figure 4a summarizes the layer-dependent exciton binding energies of other TMDs, including 3D semiconductors, described in the literature. The reported experimental exciton binding energies of monolayer TMD materials are quite high and widely distributed, whereas those of thin 2D TMDs differ fundamentally from those of 3D semiconductors. The strong dielectric screening in 3D semiconductors allows the wave function to extend over a few to several nanometers, whereas the poor dielectric screening in 2D TMDs causes a significant increase in the exciton binding energy. The exciton binding energy is also closely related to SOC (Fig. 4b). The density-functional calculations for monolayer TMDs illustrate that the exciton binding energy reduces at high values of *Δ*_SO_—it reduces 710 meV for 2H-MoTe_2_, smaller than those for 2H-MoS_2_ and 2H-MoSe_2_; the value of *Δ*_SO_ for 2H-MoTe_2_ is approximately 300 meV, which is larger than for others^16^. A similar trend is theoretically observed in multilayered TMDs. In contrast, experimentally, the situation for multilayered TMDs is different. We note that the large discrepancy between the experimental and calculated *Δ*_SO_ dependent-exciton binding energies of 2H-MoTe_2_ is not known yet. The exciton binding energy of approximately 300 meV and *Δ*_SO_ of approximately 325 meV in multilayered 2H-MoTe_2_ are unusually large, whereas relatively low values are observed in multilayered 2H-MoS_2_ and 2H-MoSe_2_. It is also worth noting that we applied the same approach for analyzing the measured spectra, which were obtained the same experimental technique. Therefore, the relative differences between TMD materials were quite reliable.

To rationalize the origin of the unusually large exciton binding energy in multilayered 2H-MoTe_2_, we introduce the *Wannier–Mott* exciton to examine the radii of excitons. For two-dimensional materials, the binding energy of a Wannier exciton can be written as $E\left(n\right)=-\frac{\frac{\mu }{{m}_{e}{\varepsilon }_{r}^{2}}{R}_{H}}{{\left(n-\frac{1}{2}\right)}^{2}}$, where ${R}_{H}$ is the Rydberg constant (13.6 eV), ${\varepsilon }_{\mathrm{r}}$ is the static relative dielectric constant, and $\mu =({m}_{\mathrm{e}}^{*}{m}_{\mathrm{h}}^{*})/({m}_{\mathrm{e}}^{*}+{m}_{\mathrm{h}}^{*})$ is the reduced mass of the electron and hole; ${m}_{\mathrm{e}}^{*}$ and ${m}_{\mathrm{h}}^{*}$ are the effective masses of the electron and hole, respectively^53^. In terms of exciton radius, ${r}_{\mathrm{n}}=\left(\frac{{{m}_{\mathrm{e}}\varepsilon }_{\mathrm{r}}{a}_{\mathrm{H}}}{\mu }\right){n}^{2}$, where ${a}_{H}$ is the Bohr radius ($5.29\times {10}^{-11} \mathrm{m}$)^54^, the exciton binding energy can also be written as $E\left(n\right)= -\frac{{n}^{2}}{{\left(n-\frac{1}{2}\right)}^{2}}\frac{{R}_{\mathrm{H}}{a}_{\mathrm{H}}}{{\varepsilon }_{\mathrm{r}}{r}_{\mathrm{n}}}$.

The exciton radii of monolayer TMDs obtained from the hydrogenic Rydberg model are presented in Supplementary Information Table S3. These are smaller than 10 Å, indicating strong exciton confinement within the layer. Together with a relatively low dielectric constant, monolayer TMDs are expected to have high exciton binding energies owing to strong Coulomb interaction or reduced dielectric screening. The discrepancy in the exciton binding energies among different monolayers is reflected by the dielectric constant and exciton radius. The exciton radii of multilayered TMDs are summarized in Table 2. Further, we provide the real parts of optical conductivities and fits for 4 nm thick 2H-MoS_2_ and 2H-MoSe_2_ (Supplementary Information Figure S8) as well as the dielectric functions for multilayered 2H-MoX_2_ at 8 K (Supplementary Information Figure S9). We note that exciton radius of multilayered 2H-MoTe_2_ remains small with 7 Å, similar to that of monolayer 2H-MoTe_2_ (Supplementary Information Table S3). Therefore, multilayered 2H-MoTe_2_ is expected to have large exciton binding energy, which is slightly reduced comparing to that of monolayer 2H-MoTe_2_ owing to its high dielectric constant (Fig. 4c). In contrast, the exciton radii of multilayered 2H-MoS_2_ and 2H-MoSe_2_ are approximately 112.55 and 24.74 Å, respectively. In these cases, the Coulomb interaction is largely screened off by the adjacent layers, leading to smaller exciton binding energies by one order of magnitude when compared with 2H-MoTe_2_. Further, we expect that *Δ*_SO_ should be also correlated with the exciton binding energy. As shown in Fig. 4b, *Δ*_SO_ of multilayered 2H-MoTe_2_ is approximately 330 meV, larger than 220 and 150 meV for multi-layered 2H-MoSe_2_ and 2H-MoS_2_, respectively. This high spin–orbit splitting energy of 2H-MoTe_2_ is expected to affect the exciton binding energy. For TMDs, *Δ*_SO_ strongly relies on the mass of the chalcogen atoms. Heavy atoms lead to large value of *Δ*_SO_.

**Table 2:** Static dielectric constant, exciton binding energy, and exciton radius of multilayered 2H-MoX_2_ at 8 K.

	4 nm MoS_2_	4 nm MoSe_2_	4 nm MoTe_2_	2 nm MoTe_2_	10 nm MoTe_2_
*ε*_r_	10.31	14.65	16.73	15.05	21.54
*E*_b_ [meV]	24.8	79.4	280	355	238
*r*_e_ [Å]	112.55	24.74	6.143	5.386	5.613

## Conclusion

In conclusion, we measured the temperature- and thickness-dependent transmittance spectra of CVD-grown 2H-MoTe_2_. The transmittance spectra were analyzed using the transfer-matrix method to obtain the optical constants. To determine the exciton binding energy in multilayered 2H-MoTe_2_, the optical conductivity was fitted by using the Lorentz model to describe the exciton peaks and Tauc–Lorentz model to describe the indirect and direct bandgaps. The exciton binding energy of 4 nm-thick multilayered 2H-MoTe_2_ was approximately 280 meV, which varies slightly with thickness. This is unusually large, by one order of magnitude, compared to other multilayered TMD semiconductors such as 2H-MoS_2_ or 2H-MoSe_2_. This can be explained by the small exciton radius, based on the 2D Rydberg model. The exciton radius of multilayered 2H-MoTe_2_ is similar to that of monolayer 2H-MoTe_2_, whereas the exciton radii of multilayered 2H-MoS_2_ and 2H-MoSe_2_ are larger, by one order of magnitude, compared to those of monolayer 2H-MoS_2_ and 2H-MoSe_2_. Consequently, multilayered 2H-MoTe_2_ exhibits intralayer Coulomb interaction, in contrast with the strong interlayer screening exhibited by 2H-MoS_2_ or 2H-MoSe_2_. Therefore, we anticipate that multilayered 2H-MoTe_2_ can be used not only for fundamental studies on many-body effects and spin/valley Hall effects but also for exciton–polariton condensates and possible applications such as room-temperature or high-temperature polariton lasing due to large exciton binding energy which stabilizes the excitons at 300 K or above 300 K^10–12^.

## Methods

### Synthesis of 2H-MoTe_2_ films

To synthesize semiconducting 2H-MoTe_2_ thin films, 0.5, 1.3, and 4 nm Mo thin films were deposited on a 300-nm-thick SiO_2_/Si substrate using a sputter system with a high basal pressure of approximately 10^–9^ Torr. The prepared Mo substrate was mounted on a two-zone CVD system with Te source. A ceramic boat with a tellurium pellet of 0.5 g (Sigma-Aldrich) was placed in the first furnace zone (zone 1) and the Mo deposited on the SiO_2_/Si substrate was placed in the second furnace zone (zone 2) to separately control the temperatures of zones 1 and 2. After purging 1000 sccm of Ar gas for 30 min, zones 1 and 2 were heated up simultaneously with the ramping rate of zone 1 first reaching 495 °C in 11 min and then zone 2 reaching 650 °C in 15 min. During the growth process, the Ar and hydrogen (H_2_) gases were introduced at a flow rate of 50 and 4 sccm, respectively, to reduce the native oxide on the surface of Mo. When the temperature of zone 2 reached 650 °C, growth was allowed for 6 h. After growth, zone 1 was cooled down by opening the chamber. Thereafter, zone 2 was opened for 30 s, followed by cooling down zone 1. During the cooling process, 500 and 4 sccm of Ar and H_2_ gases, respectively, were flown to remove the reactants. Consequently, 2, 4, and 10 nm thick 2H-MoTe_2_ films were successfully synthesized from 0.5, 1.3, and 4 nm Mo thin films, respectively.

### Transfer of 2H-MoTe_2_ onto the quartz substrate

To study the electronic properties of 2H-MoTe_2_ using optical spectroscopy, the 2H-MoTe_2_ films on SiO_2_/Si substrate were transferred onto a quartz substrate using the conventional polymethylmethacrylate (PMMA) method. Further details can be found in our previous report^44^.

### Optical measurement (FTIR)

FTIR spectroscopy is an experimental technique used to obtain the absorbance spectra in a wide infrared range from far-infrared to visible. To measure the transmittance spectra of our samples, we used a commercial FTIR spectrometer (Bruker Vertex 80v). As Vertex 80v is a vacuum-type spectrometer, the measured power spectrum is free from moisture and air absorptions. The spectrometer consists of three major components: a tungsten lamp source, a CaF_2_ beam splitter, and an InGaAs detector for the near-infrared range (4000–12,000 cm^−1^ or 0.5–1.5 eV) and an Si-diode detector for the visible range (8000–25,000 cm^−1^ or 1.0–3.0 eV). We used a commercial cold finger-type Advanced Research Systems (ARS) optical cryostat and a commercial temperature controller (Lakeshore 325) to control the sample temperature, both below and above room temperature. We used liquid helium as a coolant and were able to control the sample temperature in the range 8–350 K.

### Fitting strategy of optical conductivity with Lorentz and Tauc-Lorentz models

We first fitted the Lorentzian exciton modes and then fitted the indirect and direct bandgap absorptions with Tauc-Lorentzian modes by adjusting the intensities of the exciton modes. The peak positions and widths of the exciton modes are unambiguously determined. We constrained that the amplitudes of the exciton modes with the same origin were almost the same. We also constrained that the maximum of the indirect bandgap absorption was located in between two peaks of the A exciton and the direct transition at K-point. Additionally, the peak near 1.8 eV consists of two components: one is the peak of the exciton A’ and the other is associated with the maximum of the direct transition at K-point. Once we got a good fit for a spectrum at 8 K then we could get reliable fits for spectra at other temperatures by a systematic adjustment of the fitting parameters. For more in detailed description, refer to Supplementary Information. It is worth noting that the absorption modes including the exciton modes were based on the calculated electronic band structure. Particularly, near the bandgap, we used a minimal number of modes: The four exciton modes and the indirect and direct bandgaps.

## Supplementary Information

Supplementary Information.
