# Supplementary material for: Unusually large exciton binding energy in multilayered 2H-MoTe2
Source: Sci Rep. 2022 Mar 16;12:4543. doi: 10.1038/s41598-022-08692-1 (PMC8927107; doi:10.1038/s41598-022-08692-1)
Supplement: Supplementary file 1 — Supplementary Information. [file 41598_2022_8692_MOESM1_ESM.pdf]

Supplementary Information

Unusually Large Exciton Binding Energy in Multilayered 2H-$MoTe_2$

Eilho Jung,[†,‡,⊥] Jin Cheol Park,[†,§,⊥] Yu-Seong Seo,[‡] Ji-Hee Kim,[†,§*] Jungseek Hwang,[‡*] and Young Hee Lee[†,‡,§*]

[†]*Center for Integrated Nanostructure Physics (CINAP), Institute for Basic Science (IBS), Sungkyunkwan university (SKKU), Suwon 16419, Republic of Korea.*

[‡]*Department of Physics, Sungkyunkwan University (SKKU), Suwon 16419, Republic of Korea*

[§]*Department of Energy Science, Sungkyunkwan University (SKKU), Suwon 16419, Republic of Korea.*

[⊥]These authors contributed equally to this work.

[*]Email address: kimj@skku.edu, jungseek@skku.edu, leeyoung@skku.edu (Y.H.L)

Our analysis consists of *two fitting processes*: One is for obtaining the optical conductivity from the measured transmittance spectra by using the transfer matrix method and the other is for obtaining the characteristic absorption modes including exciton modes, indirect and direct bandgaps from the optical conductivity by using the Lorentz and Tauc-Lorentz models. The followings are detailed descriptions for those two separated fitting processes.

Optical conductivity obtained from measured transmittance using transfer matrix method

We used the transfer matrix method[1,2] to obtain the optical properties of each layer from the measured optical spectra of a thin multilayered sample. The information on the optical properties (or constants) of each layer is contained in the transfer matrix[1]. Because all possible the multi-reflections at all the interfaces are included in the transfer matrix method, reliable optical properties (or constants) of each layer can be obtained using this method. Detailed theoretical description of the transfer matrix method can be found in previous literature[1-3].

In our case, each sample consists of two layers: Thin transition metal dichalcogenide (TMD) film and quartz substrate. To obtain the optical conductivity of the thin film and substrate, one should measure, at least, two set of transmittance spectra: Bare substrate and TMD film/substrate (see Figure 1d). We analyzed the measured transmittance spectra of bare substrate and each sample (TMD film/substrate) by using the transfer matrix method. First, we fitted the transmittance spectrum of bare quartz substrate (see Figure 1d) and obtained the fitting parameters for the substrate layer. Then, we fitted the transmittance spectrum of the TMD film/substrate using the fitting parameters of the substrate layer and additional fitting parameters for the TMD film layer (see Figure 1d). We note that, for the fitting to the transmittance of TMD/substrate, we fixed the fitting parameters of the substrate and adjusted the fitting parameters for the TMD layer. Therefore, in this way, we obtained two sets of fitting parameters for the two layers, separately. To achieve a good-quality fit to the transmittance

spectrum, we used many Lorentz modes and employed a least-squares procedure to adjust the fitting parameters of the Lorentz modes for each layer (see Table 1 in Ref. [3]). Using the resulting fitting parameters of the TMD layer, we obtained the optical conductivity of TMD film (as shown in Figure 2a); usually, the obtained optical conductivity is a smooth curve. We note that the Lorentz modes used here should be discriminated from the Lorentz modes used for fitting the optical conductivity with the Lorentz and Tauc-Lorentz models. Here, we used many Lorentz modes to achieve as good quality of fits as we can (see Figure 1d).

Fitting procedure of optical conductivity using Lorentz and Tauc-Lorentz models

In Figure 2c, we show the optical conductivity data obtained from the measured transmittance spectra using the transfer matrix method and the corresponding fits obtained by using the two models: Lorentz and Tauc–Lorentz (TL) models. The Lorentz model can be used to describe symmetric exciton absorptions (electron-hole pairs), whereas the TL model can be used to describe asymmetric direct and indirect bandgap absorptions. To obtain a reliable fit to the optical conductivity by using the Lorentz and TL model, the following several factors are considered. The absorption modes in this analysis were based on the theoretically calculated band structure of multilayer 2H-$MoTe_2$, of which schematic is shown in Figure 2b.

The positions and widths of Lorentz modes for A and B (A' and B') excitons are unambiguously determined from the measured spectra. We assumed that the strengths (A_{i}) of the Lorentz modes of the A and B (A' and B') excitons are similar to each other because they originate from a single exciton level. Based on the schematic band structure of multilayer 2H-$MoTe_2$, we constrained that the exciton levels at the K-point and Γ-point are lower than the conduction bands at the K-point and Γ-point, respectively. In the TL mode, there are two positional parameters of the bandgap $(E_{\mathrm{g,j}})$ and the absorption maximum (ω_{j}). We also constrained that ω_{j} of the indirect transition is smaller than $E_{\mathrm{g,j}}$ of the K-point direct

transition. Also, ω_j of the K-point direct transition is smaller than $E_{\mathrm{g,j}}$ of the Γ-point direct transition. Beyond 2 eV, the fitting is further improved by adding two unknown Lorentz components (X and X'). We note that X and X' are not new; they were observed and denoted as ϕ and C in previous literature[4]. The energy difference between the conduction band minimum and the exciton level is defined as the exciton binding energy. We fitted the optical conductivities with Lorentz and Tauc-Lorentz modes at various temperatures between 8 and 350 K as shown Figure S2, S3, and S4.

Interestingly, there is a narrow energy region that we could not fit well; the region is in between A and B excitons (see Fig. 2c). To get a better fit in this region, we need to add an additional peak, α as shown in Figure S6. This additional α peak can be assigned as the 2s state of the A exciton. The strength of the α peak increases as the film thickness increases. In Figure S6, we also show the transmittance spectrum $(T(E) = T_{\mathrm{film+sub}}/T_{\mathrm{sub}})$ and the second derivative of the transmittance $(d^2T(E)/dE^2)$, where E is the energy. In the second derivative, we observe a small peak near 1.2 eV. The peak in the second derivative may be related to the α peak.

Supplementary Figures

S1. Atomic force microscopy (AFM) images of 2H-$MoTe_2$ film on SiO_2/Si substrate.

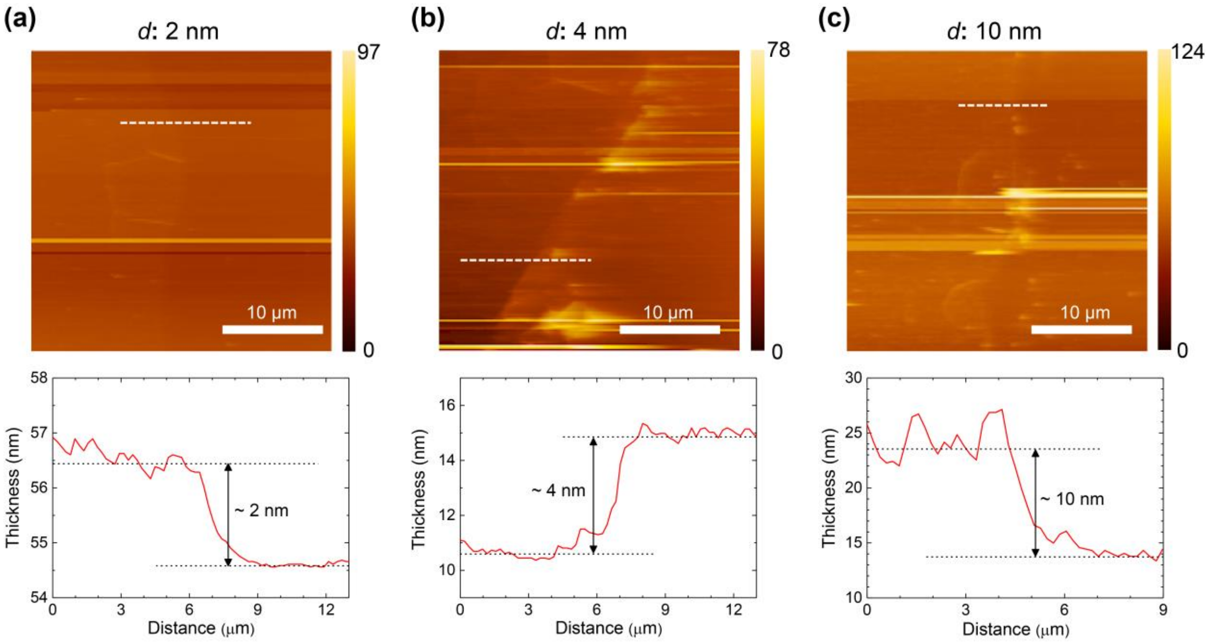

Figure S1. AFM images for (a) 2 nm thick 2H-$MoTe_2$, (b) 4 nm thick 2H-$MoTe_2$, and (c) 10 nm thick 2H-$MoTe_2$. Each plot shows corresponding height profile to estimate thickness of 2H-$MoTe_2$.

S2. Real part of optical conductivity and Lorentz and TL model fits for 2 nm thick 2H-$MoTe_2$ at various temperatures.

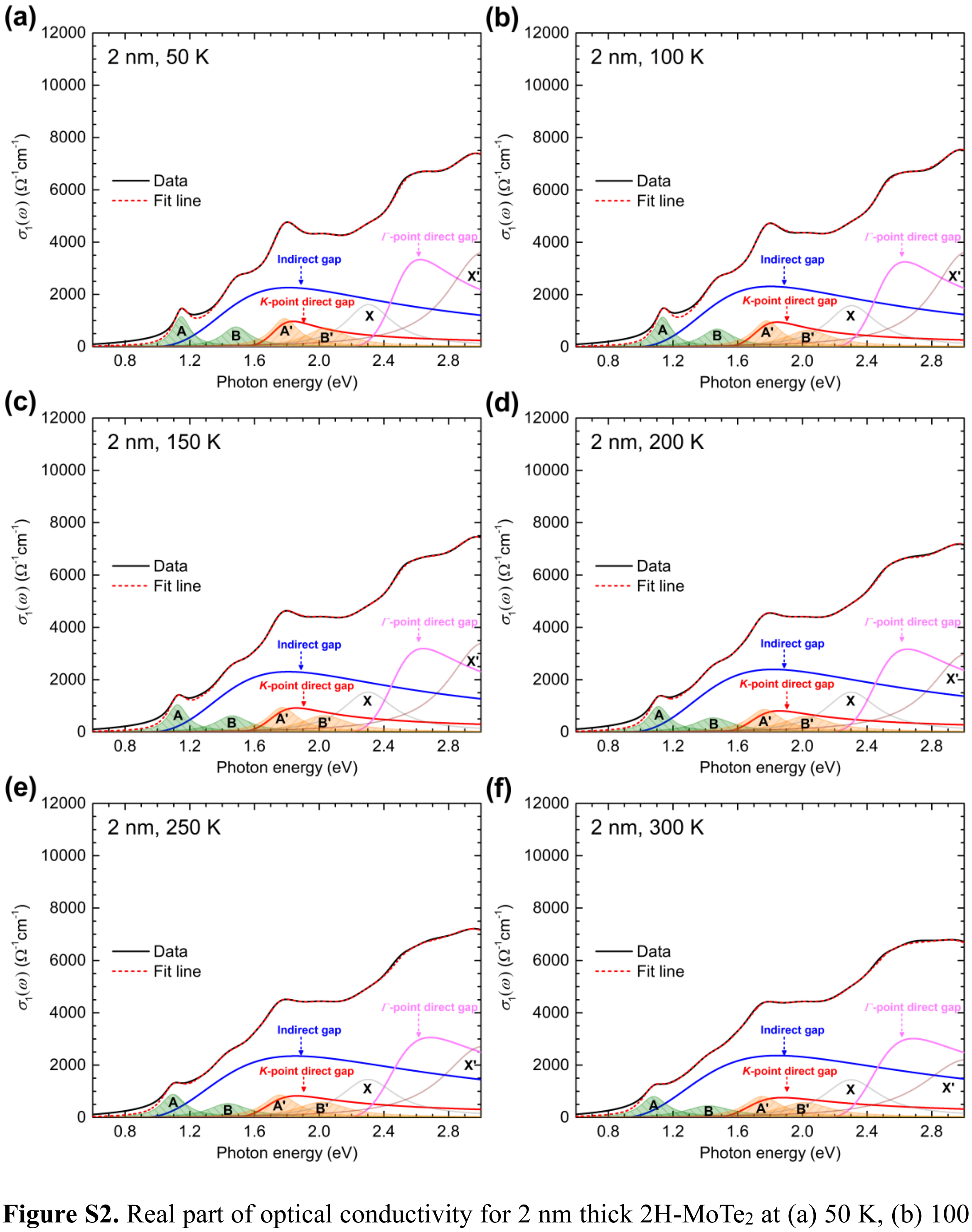

Figure S2. Real part of optical conductivity for 2 nm thick 2H-$MoTe_2$ at (a) 50 K, (b) 100 K, (c) 150 K, (d) 200 K, (e) 250 K, and (f) 300 K. All figures are fitted using Lorentz and TL models.

S3. Real part of optical conductivity and Lorentz and TL model fits for 4 nm thick 2H-$MoTe_2$ at various temperatures.

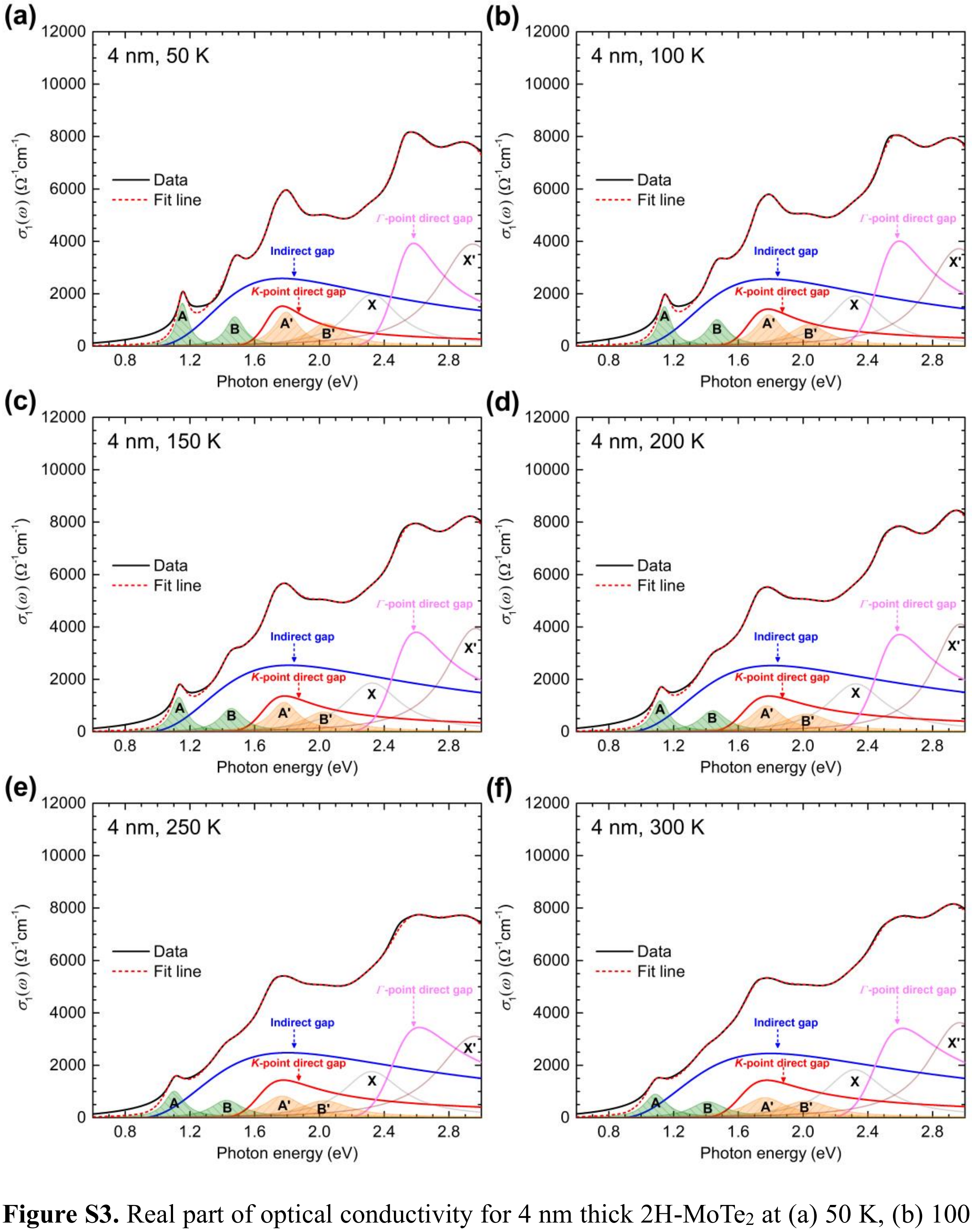

Figure S3. Real part of optical conductivity for 4 nm thick 2H-$MoTe_2$ at (a) 50 K, (b) 100 K, (c) 150 K, (d) 200 K, (e) 250 K, and (f) 300 K. All figures are fitted using Lorentz and TL models.

S4. Real part of optical conductivity and Lorentz and TL model fits for 10 nm thick 2H-$MoTe_2$ at various temperatures.

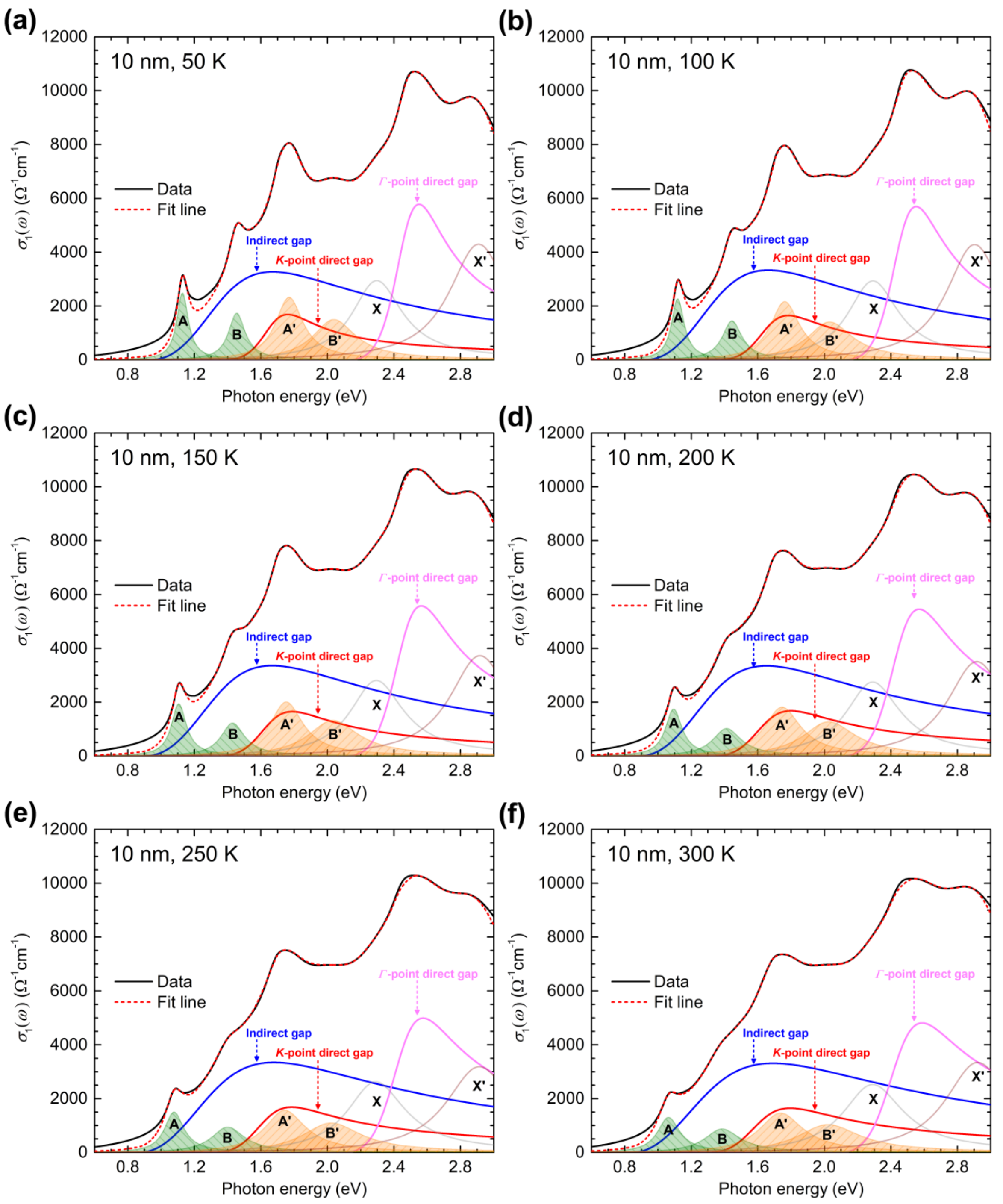

Figure S4. Real part of optical conductivity for 10 nm thick 2H-$MoTe_2$ at (a) 50 K, (b) 100 K, (c) 150 K, (d) 200 K, (e) 250 K, and (f) 300 K. All figures are fitted using Lorentz and TL models.

S5. Real part of optical conductivity and fit with four Lorentz and three TL modes in 2H-$MoTe_2$.

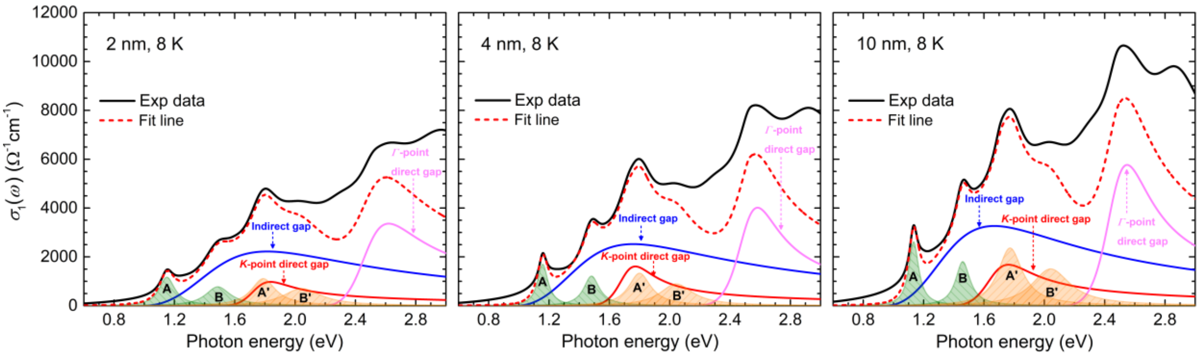

Figure S5. Fits of real part of optical conductivity with four Lorentz and three TL modes for 2, 4, and 10 nm thick 2H-$MoTe_2$ at 8 K.

S6. Temperature- and thickness-dependent width and position of A and B excitons of 2H-$MoTe_2$

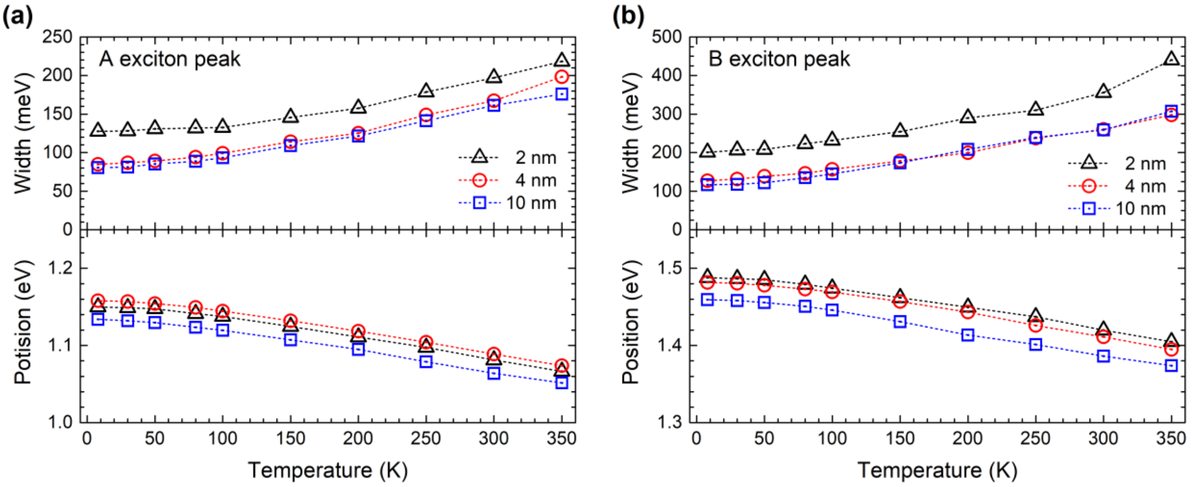

Figure S6. The widths and positions of A and B excitons of 2H-$MoTe_2$ as functions of temperature at three different thicknesses (2, 4, and 10 nm).

S7. Transmittance spectra and their second derivatives of 2H-$MoTe_2$ samples.

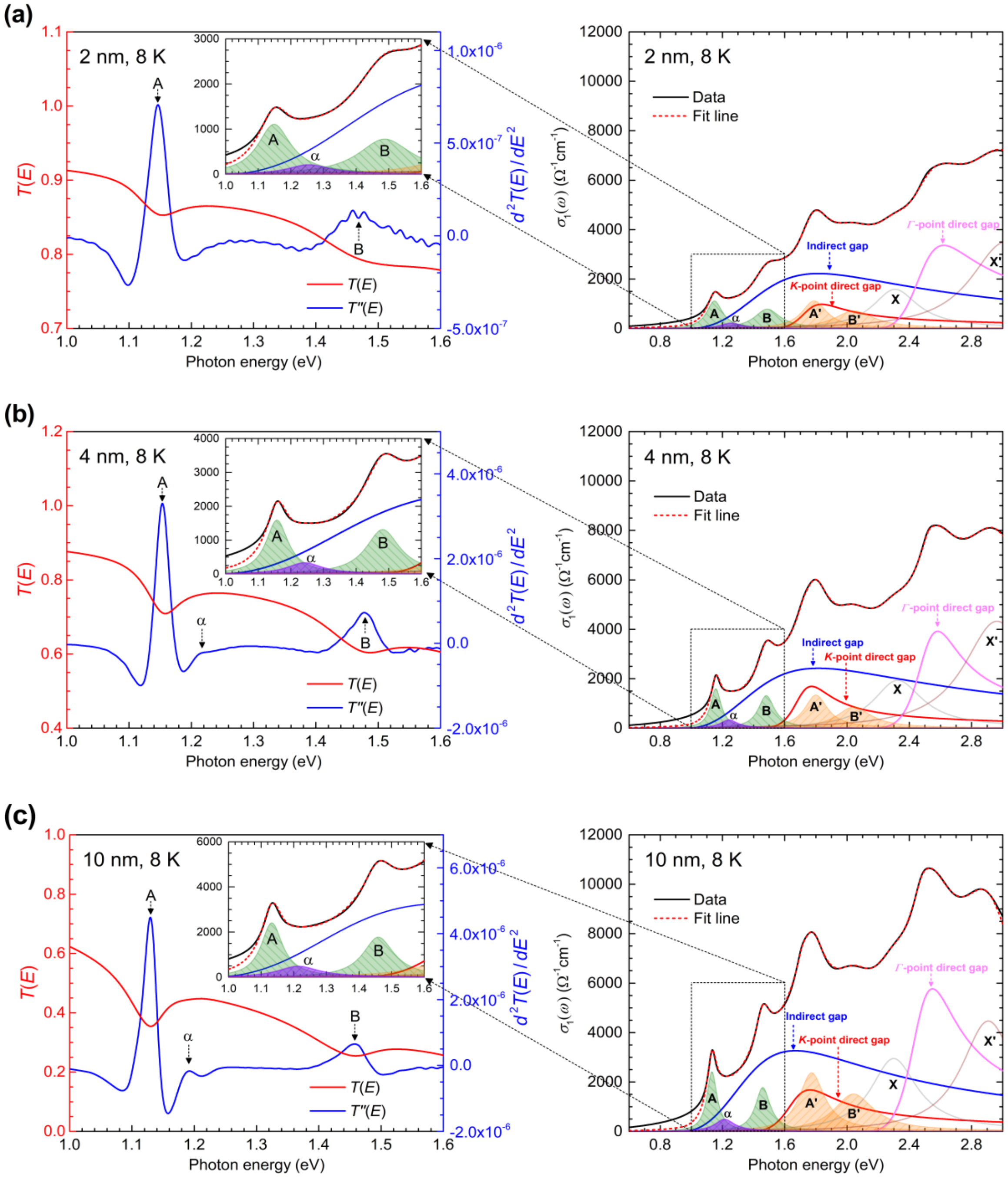

Figure S7. The transmittance spectra ($T_{\text{film+sub}}/T_{\text{sub}}$, red solid line) and their second derivatives ($d^2T(E)/dE^2$, blue solid line) for (a) 2 nm thick, (b) 4 nm thick, and (c) 10 nm thick 2H-$MoTe_2$ at 8 K. The second derivative spectrum clearly shows a peak (α peak) located between A and B excitons. The insets and panels on the right show the optical conductivity data and fits including the α peak.

S8. Data and Fits for 4 nm thick 2H-MoS_2 and 2H-$MoSe_2$ at 8 K.

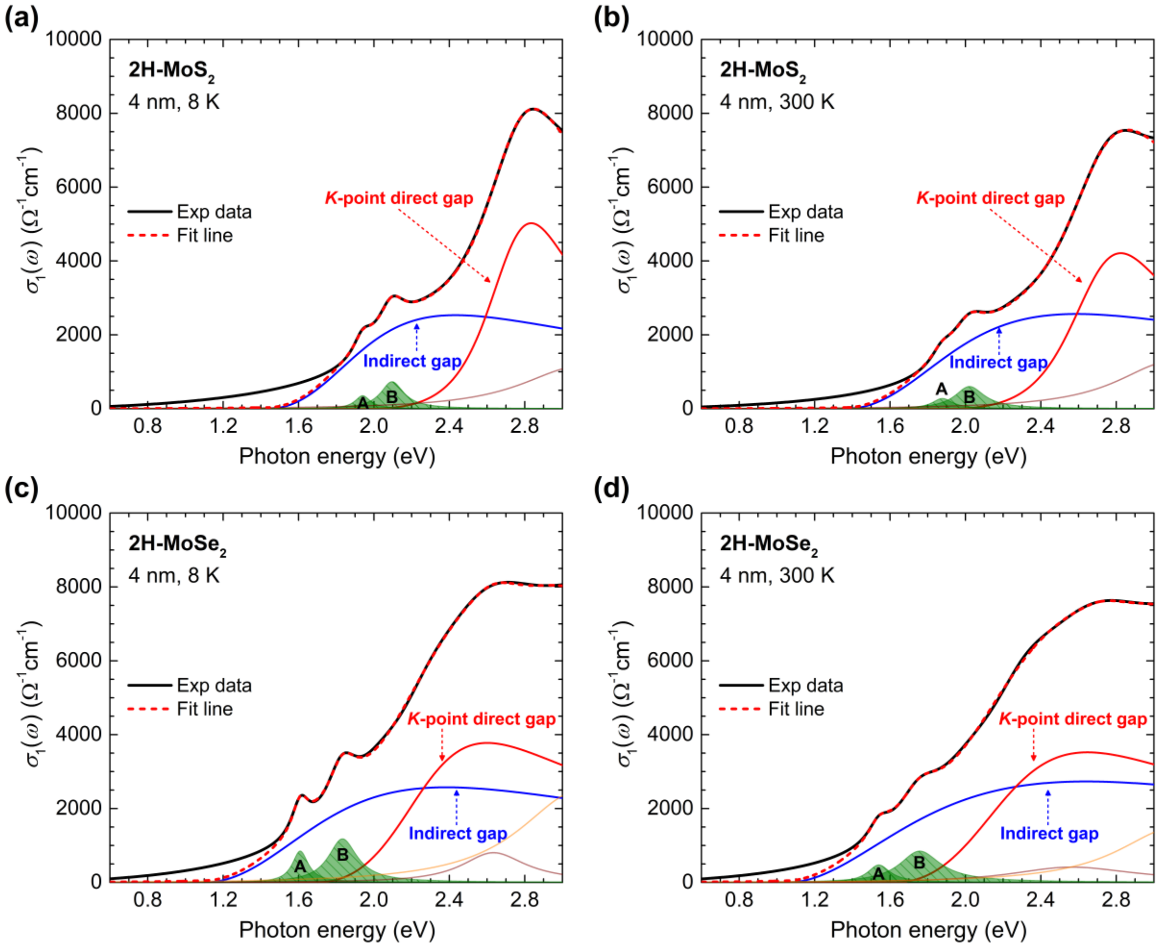

Figure S8. Real part of optical conductivity for 4 nm thick 2H-MoS_2 and 2H-$MoSe_2$ at (a) 8 K, (b) 300 K for 2H-MoS_2, (c) 8 K, (d) 300 K for 2H-$MoSe_2$. All figures are fitted using Lorentz and TL models.

S9. Dielectric functions of multilayered 2H-MoX_2

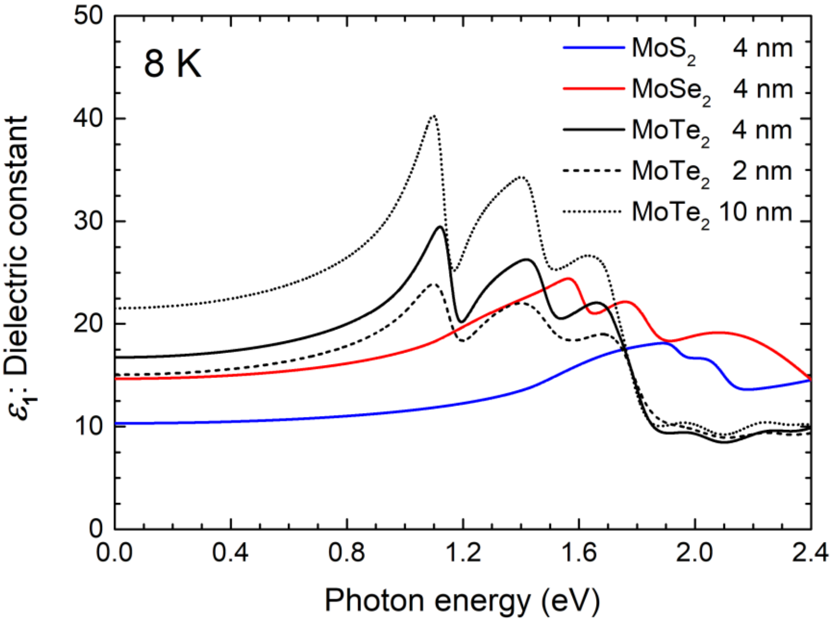

Figure S9. Real part of dielectric functions of multilayered 2H-MoX_2 at 8 K.

Table S1. Exciton binding energy (E_b) and spin-orbit splitting energy (Δ_{SO}) in monolayer and bilayer TMDs

Materials	E_b [eV]	Δ_{SO} [meV]
**MoS_2**	1.03 (monolayer at calculation) [5]<br>0.54 (monolayer at calculation) [6]<br>0.64 ± 0.08 (monolayer at 300 K) [7]<br>≥ 0.57 (monolayer at 300 K) [8]<br>0.58 (monolayer at 4 K) [9]	180 (monolayer at calculation) [5]
**$MoSe_2$**	0.91(monolayer at calculation) [5]<br>0.47 (monolayer at calculation) [6]<br>0.59 (monolayer at 10 K) [10]<br>0.51 (monolayer at 4 K) [9]<br>0.21 (bilayer at 10 K) [10]	250 (monolayer at calculation) [5]
**$MoTe_2$**	0.71 (monolayer at calculation) [5]<br>0.50 (monolayer at calculation) [11]<br>0.58 ± 0.08 (monolayer at 300 K) [12]	300 (monolayer at calculation) [5]
**WS_2**	1.04 (monolayer at calculation) [5]<br>0.50 (monolayer at calculation) [6]<br>0.83 (monolayer at 4 K) [13]<br>0.32 ± 0.05 (monolayer at 300 K) [7]<br>0.16 (monolayer at 240 K) [14]<br>0.08 (bilayer at 230 K) [14]	440 (monolayer at calculation) [5]
**WSe_2**	0.90 (monolayer at calculation) [5]<br>0.45 (monolayer at calculation) [6]<br>0.79 (monolayer at 4 K) [13]<br>0.72 (monolayer at 77 K) [10]<br>0.23 (bilayer at 77 K) [10]	480 (monolayer at calculation) [5]

Table S2. Exciton binding energy (E_b) and spin-orbit splitting energy (Δ_{SO}) in 3D bulk semiconductors and multilayered TMDs

Materials	E_b [meV]	Δ_{SO} [meV]
**Si**	10 (bulk at 212 K) [15]	-
**Ge**	2.7±0.4 (bulk at 90 K) [15]	-
**GaAs**	3.4 (bulk at 21 K) [16]	-
**MoS_2**	84 (100 nm at 13 K) [17]<br>50 (bulk at 5 K) [18]<br>24.8 (4 nm at 8 K) [this work]	200 (bulk at 5 K) [18]<br>161 (bulk at 300 K) [19]<br>258 (bulk at calculation) [19]
**$MoSe_2$**	67 (bulk at 5 K) [18]<br>79.4 (4 nm at 8 K) [this work]	270 (bulk at 5 K) [18]<br>175 (bulk at 300 K) [19]<br>294 (bulk at calculation) [19]
**$MoTe_2$**	150 (40 nm at calculation) [20]<br>70 (bulk at calculation) [21]<br>355 (2nm at 8 K) [this work]<br>280 (4 nm at 8 K) [this work]<br>238 (10 nm at 8 K) [this work]	310 (bulk at 5 K) [18]<br>238 (bulk at 300 K) [19]<br>331 (bulk at calculation) [19]
**WS_2**	-	-
**WSe_2**	56 (bulk at 1.8 K) [22]<br>50 (bulk at 5 K) [18]	480 (bulk at 5 K) [18]

Table S3. Static dielectric constant and exciton radius of monolayer MoX_2

	**MoS_2**	**$MoSe_2$**	**$MoTe_2$**
**ε_r**	4.26 [5]	4.74 [5]	5.76 [5]
**r_e [Å]**	6.6 [a)]	6.7 [a)]	7.0 [a)]

a) These values were calculated from ε_r based on ref. 5.

REFERENCES

1 Fowles, G. R. & Lynch, D. W. Introduction to Modern Optics. *Am. J. Phys.* **36**, 770-771,(1968) doi:10.1119/1.1975142.

2 Jo, J., Jung, E., Park, J. C. & Hwang, J. Comparative study of optical analysis methods for thin films. *Curr. Appl. Phys.* **20**, 237-243,(2020) doi:10.1016/j.cap.2019.11.010.

3 Hwang, J., Tanner, D. B., Schwendeman, I. & Reynolds, J. R. Optical properties of nondegenerate ground-state polymers: Three dioxythiophene-based conjugated polymers. *Phys. Rev. B* **67**,(2003) doi:10.1103/PhysRevB.67.115205.

4 Bromley, R. A., Murray, R. B. & Yoffe, A. D. The band structures of some transition metal dichalcogenides. III. Group VIA: trigonal prism materials. *J. Phys. C: Solid State Phys.* **5**, 759-778,(1972) doi:10.1088/0022-3719/5/7/007.

5 Ramasubramaniam, A. Large excitonic effects in monolayers of molybdenum and tungsten dichalcogenides. *Phys. Rev. B* **86**, 115409,(2012) doi:10.1103/PhysRevB.86.115409.

6 Berkelbach, T. C., Hybertsen, M. S. & Reichman, D. R. Theory of neutral and charged excitons in monolayer transition metal dichalcogenides. *Phys. Rev. B* **88**, 045318,(2013) doi:10.1103/physrevb.88.045318.

7 Hill, H. M. *et al.* Observation of Excitonic Rydberg States in Monolayer MoS_2 and WS_2 by Photoluminescence Excitation Spectroscopy. *Nano Lett.* **15**, 2992-2997,(2015) doi:10.1021/nl504868p.

8 Klots, A. R. *et al.* Probing excitonic states in suspended two-dimensional semiconductors by photocurrent spectroscopy. *Sci. Rep.* **4**, 6608,(2014) doi:10.1038/srep06608.

9 Wang, G. *et al.* Exciton states in monolayer $MoSe_2$: impact on interband transitions. *2D Mater.* **2**, 045005,(2015) doi:10.1088/2053-1583/2/4/045005.

10 Liu, H. J. *et al.* Molecular-beam epitaxy of monolayer and bilayer WSe_2: a scanning tunneling microscopy/spectroscopy study and deduction of exciton binding energy. *2D Mater.* **2**, 034004,(2015) doi:10.1088/2053-1583/2/3/034004.

11 Robert, C. *et al.* Excitonic properties of semiconducting monolayer and bilayer $MoTe_2$. *Phys. Rev. B* **94**, 155425,(2016) doi:10.1103/PhysRevB.94.155425.

12 Yang, J. *et al.* Robust Excitons and Trions in Monolayer $MoTe_2$. *ACS Nano* **9**, 6603-6609,(2015) doi:10.1021/acsnano.5b02665.

13 Hanbicki, A. T., Currie, M., Kioseoglou, G., Friedman, A. L. & Jonker, B. T.

Measurement of high exciton binding energy in the monolayer transition-metal dichalcogenides WS_2 and WSe_2. *Solid State Commun.* **203**, 16-20,(2015) doi:10.1016/j.ssc.2014.11.005.

14 Jo, S., Ubrig, N., Berger, H., Kuzmenko, A. B. & Morpurgo, A. F. Mono- and bilayer WS_2 light-emitting transistors. *Nano Lett.* **14**, 2019-2025,(2014) doi:10.1021/nl500171v.

15 Macfarlane, G. G., McLean, T. P., Quarrington, J. E. & Roberts, V. Exciton and phonon effects in the absorption spectra of germanium and silicon. *J. Phys. Chem. Solids* **8**, 388-392,(1959) doi:10.1016/0022-3697(59)90372-5.

16 Sturge, M. D. Optical Absorption of Gallium Arsenide between 0.6 and 2.75 eV. *Phys. Rev.* **127**, 768-773,(1962) doi:10.1103/PhysRev.127.768.

17 Saigal, N., Sugunakar, V. & Ghosh, S. Exciton binding energy in bulk MoS_2: A reassessment. *Appl. Phys. Lett.* **108**, 132105,(2016) doi:10.1063/1.4945047.

18 Beal, A. R., Knights, J. C. & Liang, W. Y. Transmission spectra of some transition metal dichalcogenides. II. Group VIA: trigonal prismatic coordination. *J. Phys. C: Solid State Phys.* **5**, 3540-3551,(1972) doi:10.1088/0022-3719/5/24/016.

19 Böker, T. *et al.* Band structure of MoS_2, $MoSe_2$, and α−$MoTe_2$: Angle-resolved photoelectron spectroscopy and ab initiocalculations. *Phys. Rev. B* **64**, 235305,(2001) doi:10.1103/PhysRevB.64.235305.

20 Arora, A. *et al.* Interlayer excitons in a bulk van der Waals semiconductor. *Nat. Commun.* **8**, 639,(2017) doi:10.1038/s41467-017-00691-5.

21 Komsa, H.-P. & Krasheninnikov, A. V. Effects of confinement and environment on the electronic structure and exciton binding energy of MoS_2 from first principles. *Phys. Rev. B* **86**, 241201(R),(2012) doi:10.1103/PhysRevB.86.241201.

22 Mitioglu, A. A. *et al.* Optical Investigation of Monolayer and Bulk Tungsten Diselenide (WSe_2) in High Magnetic Fields. *Nano Lett.* **15**, 4387-4392,(2015) doi:10.1021/acs.nanolett.5b00626.
